# Adverse Event Assessment of Antimuscarinics for Treating Overactive Bladder: A Network Meta-Analytic Approach

**DOI:** 10.1371/journal.pone.0016718

**Published:** 2011-02-23

**Authors:** Thomas M. Kessler, Lucas M. Bachmann, Christoph Minder, David Löhrer, Martin Umbehr, Holger J. Schünemann, Alfons G. H. Kessels

**Affiliations:** 1 Horten Centre for Patient Oriented Research, University of Zürich, Zürich, Switzerland; 2 Neuro-Urology, Spinal Cord Injury Centre, Balgrist University Hospital, Zürich, Switzerland; 3 Department of Clinical Epidemiology and Biostatistics, McMaster University, Hamilton, Ontario, Canada; 4 Department of Clinical Epidemiology and Medical Technology Assessment, Maastricht University Medical Centre, Maastricht, The Netherlands; Lerner Research Institute, Cleveland Clinic, United States of America

## Abstract

**Background:**

Overactive bladder (OAB) affects the lives of millions of people worldwide and antimuscarinics are the pharmacological treatment of choice. Meta-analyses of all currently used antimuscarinics for treating OAB found similar efficacy, making the choice dependent on their adverse event profiles. However, conventional meta-analyses often fail to quantify and compare adverse events across different drugs, dosages, formulations, and routes of administration. In addition, the assessment of the broad variety of adverse events is dissatisfying. Our aim was to compare adverse events of antimuscarinics using a network meta-analytic approach that overcomes shortcomings of conventional analyses.

**Methods:**

Cochrane Incontinence Group Specialized Trials Register, previous systematic reviews, conference abstracts, book chapters, and reference lists of relevant articles were searched. Eligible studies included randomized controlled trials comparing at least one antimuscarinic for treating OAB with placebo or with another antimuscarinic, and adverse events as outcome measures. Two authors independently extracted data. A network meta-analytic approach was applied allowing for joint assessment of all adverse events of all currently used antimuscarinics while fully maintaining randomization.

**Results:**

69 trials enrolling 26′229 patients were included. Similar overall adverse event profiles were found for darifenacin, fesoterodine, transdermal oxybutynin, propiverine, solifenacin, tolterodine, and trospium chloride but not for oxybutynin orally administered when currently used starting dosages were compared.

**Conclusions:**

The proposed generally applicable transparent network meta-analytic approach summarizes adverse events in an easy to grasp way allowing straightforward benchmarking of antimuscarinics for treating OAB in clinical practice. Most currently used antimuscarinics seem to be equivalent first choice drugs to start the treatment of OAB except for oral oxybutynin dosages of ≥10 mg/d which may have more unfavorable adverse event profiles.

## Introduction

Overactive bladder (OAB) is a widespread chronic illness that affects the lives of millions of people worldwide at all ages but is more common in the elderly with a prevalence of up to 31% in women and 42% in men aged over 75 years [1]. OAB has a major impact on quality of life, affecting emotional, social, sexual, occupational, and physical aspects of daily life [2]–[4], and it is associated with a greater risk of falls and injuries, including fractures [5], which may even lead to death. Besides the debilitating manifestations for patients, OAB also imposes substantial economic burden as direct annual costs are comparable to those of other chronic diseases such as dementia and diabetes mellitus [6].

Non-surgical treatment is the mainstay of therapy for OAB including lifestyle modifications, behavioral therapy, biofeedback, bladder training, medication and a combination of these options. Antimuscarinics are the pharmacological treatment of choice for OAB. Seven antimuscarincs (darifenacin, fesoterodine, oxybutynin, propiverine, solifenacin, tolterodine, and trospium chloride) with different dosages, formulations, and routes of administration are currently used for treating OAB and all have well-established and similar efficacy (figure S1) shown in systematic reviews [7]–[13], so that the selection of the most appropriate depends on their adverse event profiles. However, over 30 different adverse events were described and only a few of them were compared systematically. Moreover, joint assessment of all reported adverse events of the currently used antimuscarinics is lacking.

In 2004 [14], the Grading of Recommendations Assessment, Development and Evaluation (GRADE) working group highlighted the importance to interpret quality of evidence in view of trade-offs between effect and side effects, or desirable and undesirable consequences of management strategies. However, conventional meta-analyses often fail to quantify and compare adverse events across different drugs, dosages, formulations, and routes of administration, because summaries usually do not use all available information on reported comparisons and assessment of the broad variety of adverse events. This is seen as a major impediment when examining the trade-offs the GRADE working group is calling for.

Recently, new meta-analytic methods – network meta-analysis – have become available which allow complete assessments across different drugs. While these methods gained some popularity in the assessment of treatment effects, their application in side effect assessment is novel. In view that all antimuscarinics show similar efficacy, the assessment of side effects alone suffices to provide the required information for decision-making and evidence grading. Therefore, we performed a network meta-analysis summarizing data from all randomized comparative clinical trials assessing adverse events of all currently used antimuscarinics. Moreover we propose a way to aggregate the broad variety of reported adverse events and finally provide adverse event charts for all assessed medications and dosages allowing straightforward benchmarking of antimuscarinics for treating OAB in clinical practice.

## Methods

### Search strategy and selection criteria

This systematic review was done according to the PRISMA statement [15]. A review protocol was elaborated, which is available on file with the authors. To identify randomized controlled trials of antimuscarinics versus placebo (Figure S2), we started with the recent Cochrane review by Nabi et al. [10] and a literature search update from June 2005 to November 2007 kindly provided by the Cochrane Incontinence Review Group according to their previously published search strategy [10]. Head-to-head comparative trials without a placebo arm were identified from the two recent meta-analysis by Chapple et al. [12] and by Novara et al. [13]. In addition, we searched reference lists and conference abstracts by hand, checked relevant reviews, book chapters, and contacted manufacturers and trialists. The search strategies are available on request.

Two investigators (TMK, DL) independently assessed reports for eligibility. To be included, studies had to be randomized controlled trials comparing at least one antimuscarinic for treating OAB with placebo or with another antimuscarinic. All currently used antimuscarinics were included, i.e. darifenacin, fesoterodine, oxybutynin, propiverine, solifenacin, tolterodine, and trospium chloride. Trials with intravesical antimuscarinic administration, drugs with less direct antimuscarinic effects (such as smooth muscle relaxants, flavoxate hydrochloride, calcium channel blockers, potassium channel openers, beta-adrenoceptor agonists, alpha-adrenoceptor antagonists, prostaglandin synthetase inhibitors, and tricyclic antidepressants), and drugs no longer used in clinical practice (such as emepronium bromide or carrageenate, dicyclomine chloride, penthienate, propantheline bromide, and terodiline) were excluded. In the case of multiple publications on the same patients, the most complete report was chosen for each trial.

### Data collection

We extracted data in duplicate (TMK, DL) and a third reviewer (LMB) resolved any discrepancies if the two reviewers disagreed. We contacted authors of eligible trials that reported insufficient data and asked them for additional information (Data S1). Dichotomous data were abstracted into 2×2 tables. For continuous data, summary estimates per group (means, changes in means) with measures of variability (standard deviation [SD], 95% confidence interval [CI]) as available were extracted.

Outcome measures were adverse events. After extraction, adverse events were classified according to the Common Terminology Criteria for Adverse Events v3.0 (CTCAE) [16] into 7 categories (gastrointestinal, ocular/visual, urinary tract related, neurological, cardiac, respiratory tract related, dermatological adverse events) and then graded using a visual analogue scale (0 = minimum severity, 10 = maximum severity) based on the consensus of 10 independent experts (Table 1). Summarizing different formulations and routes of administration, antimuscarinics were categorized based on the daily dose used into the following groups: darifenacin 3.75 mg/d, 7.5 mg/d, 15 mg/d, 30 mg/d; fesoterodine 4 mg/d, 8 mg/d, 12 mg/d; oxybutynin 5 mg/d, 7.5 mg/d, 9 mg/d, 10 mg/d, 15 mg/d, 20 mg/d; oxybutynin transdermal system (TDS) 1.3 mg/d, 2.6 mg/d, 3.9 mg/d; propiverine 20 mg/d, 30 mg/d, 45 mg/d; solifenacin 2.5, 5 mg/d, 10 mg/d, 20 mg/d; tolterodine 1 mg/d, 2 mg/d, 4 mg/d, 8 mg/d; trospium chloride 40 mg/d, 45 mg/d, 60 mg/d.

**Table 1:** Categorization and grading of adverse events.

Type of adverse events	Grading using VAS
**Gastrointestinal adverse events**	
Dry mouth	4
Dry throat	4
Dysgeusia	4
Constipation	4
Diarrhoe	4
Abdominal pain	5
Gastritis	5
Dyspepsia	4
Nausea	5
Vomitus	6
Unspecified gastrointestinal adverse events	5
**Ocular/visual adverse events**	
Dry eye	4
Blurred vision	6
**Urinary tract related adverse events**	
Urinary retention	7
Voiding difficulty	5
Dysuria	5
Urinary tract infection	6
Unspecified urinary tract related adverse events	6
**Neurological adverse events**	
Fatigue	5
Somnolence	8
Sedation	7
Insomnia	6
Confusion	7
Cognitive impairment	7
Depression/lethargy	7
Dizziness/vertigo	5
Headache	5
**Cardiac adverse events**	
Palpitation/tachycardia	5
Hypertension	6
Orthostatic disturbance	6
Fall	8
**Respiratory tract related adverse events**	
Dry nose	3
Cough	4
Nasopharyngitis	4
Sinusitis	4
Upper respiratory tract infection	6
Influenza	6
**Dermatological adverse events**	
Dry skin	2
Erythema/exanthema	4
Pruritus	5

VAS: visual analogue scale (0 = minimum severity, 10 = maximum severity).

### Statistical analysis

We aimed to produce a descriptive model allowing us summarizing data on adverse events statistically. For each of the 7 adverse event categories and each of the treatment arms, the number of adverse events, weighted according to the grading provided in Table 1, were added up and divided by the total number of patients in the corresponding treatment arm. In this way, the weighted adverse events per patient in each of the trials given a specific treatment and dosage were determined. The total score of adverse events was calculated by adding up these estimates of the 7 adverse event categories.

For all of the 8 adverse event outcomes (7 adverse event categories and the total score of adverse events), a linear regression analysis was performed with drug and dosage as covariates and using a similar concept as Berlin et al. [17] and as Hasselblad [18], the event outcome for each single treatment arm as the dependent variable. To preserve randomization within each trial, we included a dummy variable for each of the studies. This dummy variable adjusted for differences in risk profiles and study setup between trials. The drugs were entered as indicator variables and the dosages were transformed to a uniform format by giving value 1 to the currently used starting dosage and the other dosages the multiplication factor as compared with this dosage. Thus, when the currently used starting dosage was 7.5 mg/d and dosages of 3.75 mg/d, 7.5 mg/d, 15 mg/d, and 30 mg/d were investigated, these values were recoded as 0.5, 1, 2, and 4, respectively. Because every drug has its own specific dose-adverse event relation, interaction terms between drug and dosage were added. Furthermore, the analysis was weighted with the total number of patients in each treatment arm as a substitute for the inverse of the variance, and the cluster option was used to take into account that results within one trial will be correlated. We investigated the influence of several trial characteristics such as age, gender, and duration of treatment. This was done by entering them as an interaction term with treatment into the model. From this regression model we estimated the difference between adverse events of the placebo and the dosages applied in standard clinical practice (i.e. darifenacin 7.5 mg/d, 15 mg/d; fesoterodine 4 mg/d, 8 mg/d; oxybutynin 10 mg/d, 15 mg/d, 20 mg/d; oxybutynin TDS 3.9 mg/d; propiverine 30 mg/d, 45 mg/d; solifenacin 5 mg/d, 10 mg/d; tolterodine 4 mg/d; trospium chloride 40 mg/d, 60 mg/d).

All analyses were performed with Stata SE 10.1 (Copyright 1996–2010 StataCorp LP, 4905 Lakeway Drive, College Station, TX 77845 USA).

## Results

We identified 82 reports (Figure S2). Three articles [19]–[21] not specifying adverse events, one article [22] not differentiating adverse events between antimuscarinic and placebo, six articles [23]–[28] not discriminating adverse events between different fixed dosages, and three articles [29]–[31] only comparing different releases within the same antimuscarinic and dosage were excluded. Thus, we finally included 69 trials [32]–[100] including one report [66] with two different age strata (Table 2, Data S2). Most trials had a parallel design (58, 84%) and were placebo-controlled (57, 83%). Overall, the included trials enrolled 26229 patients. The mean age was 59 years (range 30 to 82), the proportion of women was 76% (range 0% to 100%) and the mean duration of treatment was 8 weeks (range 1 to 52 weeks).

**Table 2:** Characteristics of trials included in the network meta-analysis.

Author	Year	No. of patients	% of females	Mean age in years	Treatment duration in weeks	Placebo	Darifenacin in mg	Fesoterodine in mg	Oxybutynin in mg	Propiverine in mg	Solifenacin in mg	Tolterodine in mg	Trospium chloride in mg
Bono [32]	1982	16*	NA	NA	1.4	+	−	−	IR 5 3	−	−	−	−
Murray [33]	1984	25*	NA	NA	4	+	−	−	IR 5 3	−	−	−	−
Riva [34]	1984	30*	100%	51.5	3	+	−	−	IR 5 3	−	−	−	−
Zorzitto [35]	1989	18*	25%	73.9	1.1	+	−	−	IR 5 2	−	−	−	−
Moore [36]	1990	48*	100%	46.2	4	+	−	−	IR 3 3	−	−	−	−
Takayasu [37]	1990	131	NA	NA	2	+	−	−	−	IR 20 1	−	−	−
Tapp [38]	1990	31*	100%	61	2	+	−	−	IR 5 4	−	−	−	−
Stöhrer [39]	1991	55	45%	33.3	3	+	−	−	−	−	−	−	IR 20 2
Thüroff [40]	1991	115	96%	48.5	4	+	−	−	IR 5 3	−	−	−	−
Wehnert [41]	1992	10*	NA	NA	3	+	−	−	IR 5 3	IR 15 3	−	−	−
Szonyi [42]	1995	57	93%	82	6	+	−	−	IR 2.5 2	−	−	−	−
Mazur [43]	1995	185	98%	47.9	3	−	−	−	−	IR 15 1, 2, 3, 4	−	−	−
Abrams [44]	1996	67	NA	NA	2	+	−	−	−	−	−	IR 0.5, 1, 2, 4 2	−
Jonas [45]	1997	241	75%	57.8	4	+	−	−	−	−	−	IR 1, 2 2	−
Van Kerrebroeck [46]	1997	240	NA	NA	12	−	−	−	IR 5 3	−	−	IR 2 2	−
Abrams [47]	1998	293	76%	56.8	12	+	−	−	IR 5 3	−	−	IR 2 2	−
Alloussi [48]	1998	309	72%	56.6	3	+	−	−	−	−	−	−	IR 20 2
Rentzhog [49]	1998	80	76%	57.3	2	+	−	−	−	−	−	IR 0.5, 1, 2, 4 2	−
Van Kerrebroeck [50]	1998	90	47%	42	2	+	−	−	−	−	−	IR 0.5, 1, 2, 4 2	−
Drutz [51]	1999	277	77%	64.2	12	+	−	−	IR 5 3	−	−	IR 2 2	−
Madersbacher [52]	1999	366	93%	49.5	4	+	−	−	IR 5 2	IR 15 3	−	−	−
Millard [53]	1999	311	75%	60.2	12	+	−	−	−	−	−	IR 1, 2 2	−
Stöhrer [54]	1999	113	39%	29.8	2	+	−	−	−	IR 15 3	−	−	−
Cardozo [55]	2000	208	62%	46.7	3	+	−	−	−	−	−	−	IR 20 2
Dorschner [56]	2000	98	79%	67.5	4	+	−	−	−	IR 15 3	−	−	−
Jünemann [57]	2000	232	NA	NA	3	+	−	−	−	−	−	IR 2 2	IR 20 2
Serrano Brambila [58]	2000	37*	100%	51.7	6	+	−	−	IR 5 3	−	−	−	−
Jacquetin [59]	2001	251	79%	55.7	4	+	−	−	−	−	−	IR 1, 2 2	−
Malone-Lee [60]	2001	177	65%	75	4	+	−	−	−	−	−	IR 1, 2 2	−
Ulshofer [61]	2001	45	92%	51.2	4	+	−	−	−	−	−	−	IR 15 3
Van Kerrebroeck [62]	2001	1524	81%	60.3	12	+	−	−	−	−	−	IR 2 2, ER 4 1	−
Appell [63]	2001	378	83%	59.1	12	−	−	−	ER 10 1	−	−	IR 2 2	−
Malone-Lee [64]	2001	378	67%	65.1	10	−	−	−	IR 5 2	−	−	IR 2 2	−
Dmochowski [65]	2002	520	92%	61.4	12	+	−	−	TDS 1.3, 2.6, 3.9 1	−	−	−	−
Zinner [66]	2002	576	87%	51	12	+	−	−	−	−	−	ER 4 1	−
		436	74%	74	12	+	−	−	−	−	−	ER 4 1	−
Lee [67]	2002	227	77%	53	8	−	−		IR 5 2	−	−	IR 2 2	−
Dmochowski [68]	2003	361	93%	63.5	12	+	−	−	TDS 3.9 1	−	−	ER 4 1	−
Homma [69]	2003	605	70%	59.3	12	+	−	−	IR 3 3	−	−	ER 4 1	−
Diokno [70]	2003	790	100%	60	10	−	−	−	ER 10 1	−	−	ER 4 1	−
Halaska [71]	2003	357	86%	53.7	52	−	−	−	IR 5 2	−	−	−	IR 20 2
Cardozo [72]	2004	910	82%	55.8	12	+	−	−	−	−	5, 10 1	−	−
Chapple [73]	2004	225	60%	56	4	+	−	−	−	−	2.5, 5, 10, 20 1	IR 2 2	−
Chapple [74]	2004	1077	75%	57.5	12	+	−	−	−	−	5, 10 1	IR 2 2	−
Haab [75]	2004	561	85%	57	12	+	3.75, 7.5, 15 1	−	−	−	−	−	−
Khullar [76]	2004	854	100%	58.2	8	+	−	−	−	−	−	ER 4 1	−
Zinner [77]	2004	523	74%	62.3	12	+	−	−	−	−	−	−	IR 20 2
Giannitsas [78]	2004	128*	100%	56	6	−	−	−	IR 5 3	−	−	IR 2 2	−
Abrams [79]	2005	745	0%	64	12	+	−	−	−	−	−	ER 4 1	−
Abrams [80]	2005	848	61%	61	12	+	−	−	−	−	−	ER 4 1	−
Cardozo [81]	2005	72	71%	54	2	+	30 1	−	−	−	−	−	−
Nitti [82]	2005	171	NA	NA	8	+	−	4, 8, 12 1	−	−	−	−	−
Romanzi [83]	2005	450	NA	NA	12	+	15 1	−	−	−	−	IR 2 2	−
Zinner [84]	2005	61*	93%	59.9	2	+	15, 30 1	−	IR 5 3	−	−	−	−
Altan-Yaycioglu [85]	2005	52	100%	41.1	4	−	−	−	IR 5 3	−	−	IR 2 2	−
Jünemann [86]	2005	202	78%	56.3	4	−	−	−	−	IR 15 2	−	IR 2 2	−
Abrams [87]	2006	222	0%	63.6	12	+	−	−	−	−	−	IR 2 2	−
Abrams [88]	2006	42*	77%	NA	2	+	−	−	IR 5 3	IR 20 1, 15 3	−	−	−
Hill [89]	2006	439	85%	54.7	12	+	7.5, 15, 30 1	−	−	−	−	−	−
Jünemann [90]	2006	988	89%	56.1	4.6	+	−	−	−	IR 15 2, ER 30 1	−	−	−
Kaplan [91]	2006	436	0%	62.3	12	+	−	−	−	−	−	ER 4 1	−
Rackley [92]	2006	850	51%	58.5	12	+	−	−	−	−	−	ER 4 1	−
Rudy [93]	2006	658	81%	61.1	12	+	−	−	−	−	−	−	IR 20 2
Zinner [94]	2006	439	87%	59.1	12	+	15 1	−	−	−	−	−	−
Corcos [95]	2006	237	85%	60.9	4	−	−	−	ER 5, 10, 15 1	−	−	−	−
Chapple [96]	2007	1132	80%	56.6	12	+	−	4, 8 1	−	−	−	ER 4 1	−
Yamaguchi [97]	2007	1582	84%	60.2	12	+	−	−	−	IR 20 1	5, 10 1	−	−
Nitti [98]	2007	832	76%	59	12	+	−	4, 8 1	−	−	−	−	−
Staskin [99]	2007	601	85%	59.4	12	+	−	−	−	−	−	−	ER 60 1
Chapple [100]	2007	584	87%	56.7	12	−	−	−	−	−	5 1	ER 4 1	−

IR: immediate release.

ER: extended release.

TDS: transdermal system.

NA: not available.

1 once daily.

2 twice daily.

3 three times daily.

4 four times daily.

*crossover design.

**Figure 1:**
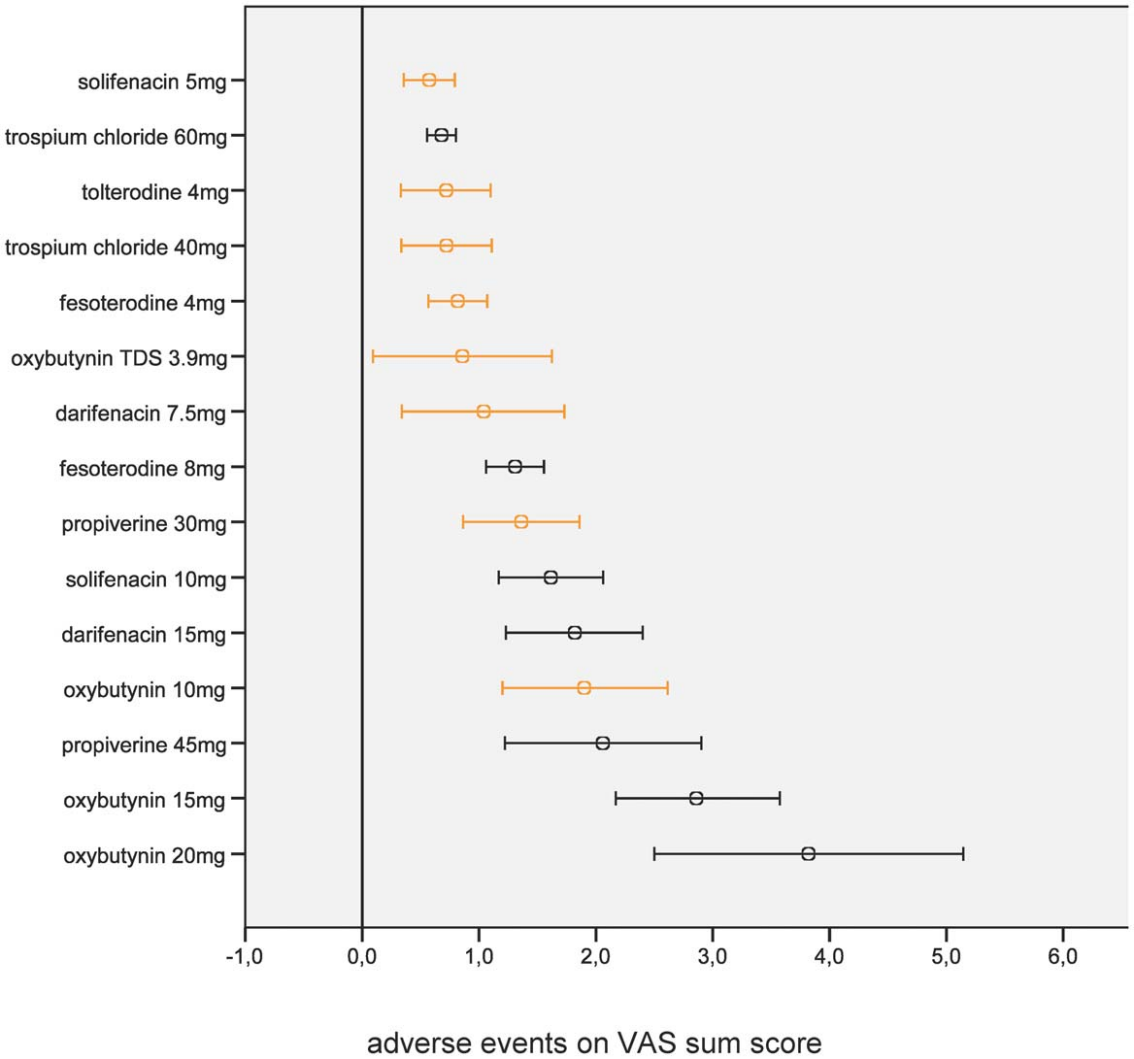
Overall adverse event profiles (from 69 trials) of different antimuscarinic treatments and dosages per day compared with placebo (reference line through 0). The orange lines represent the currently used starting dosages (oxybutynin 15 mg/d and trospium chloride 60 mg/d may also be used as starting dosages).○ mean, 95% confidence interval, TDS transdermal system, VAS visual analogue scale.

We found similar overall adverse event profiles for darifenacin, fesoterodine, transdermal oxybutynin, propiverine, solifenacin, tolterodine, and trospium chloride but not for oxybutynin orally administered when currently used starting dosages were compared (Figure 1). Orally administered oxybutynin dosages of ≥10 mg/d demonstrated the worst adverse event profiles. Darifenacin, fesoterodine, oxybutynin orally administered, propiverine, and solifenacin showed a positive and significant dose-adverse event relation.

**Figure 2:**
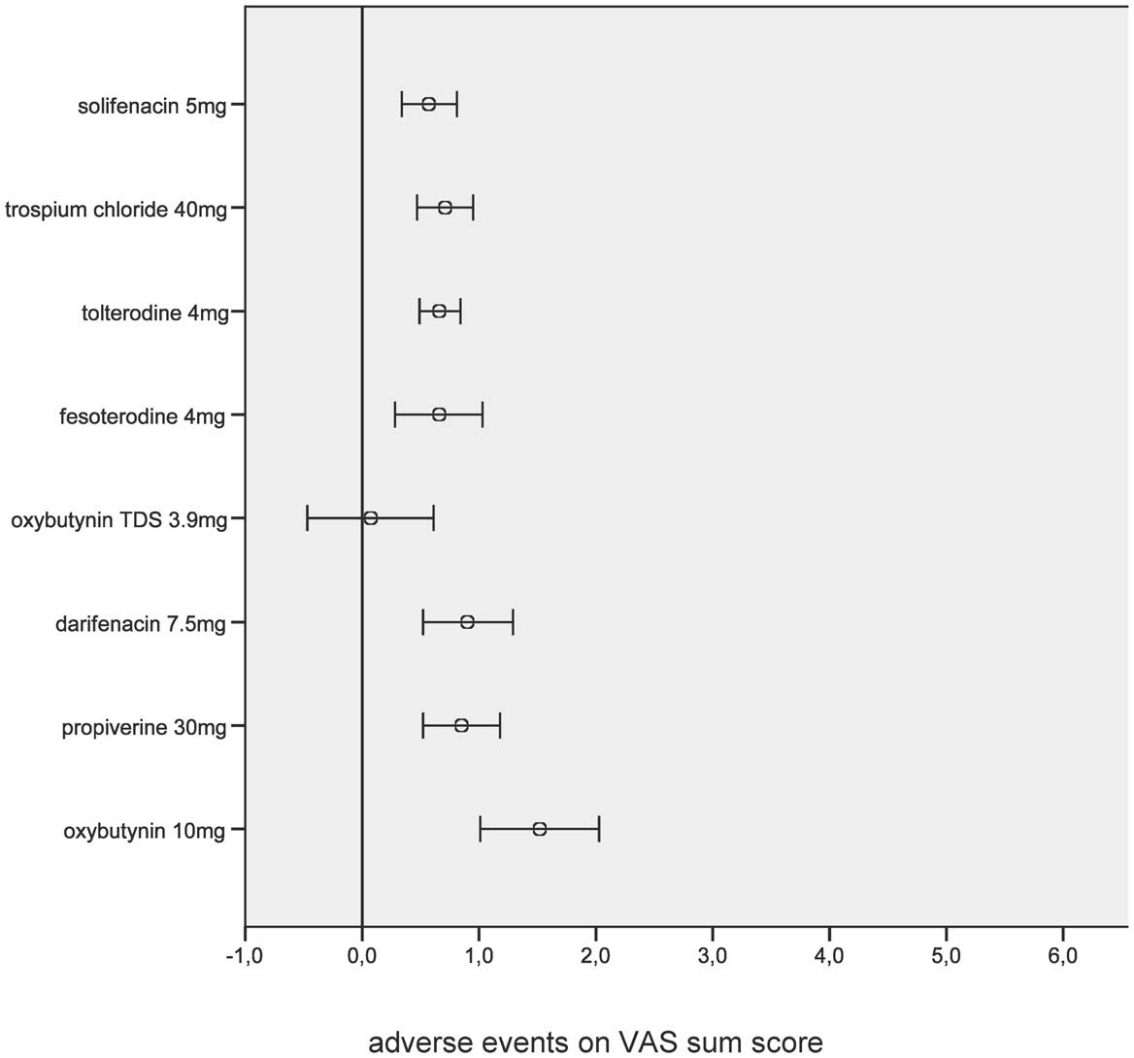
Gastrointestinal adverse events profiles (from 69 trials) of different antimuscarinic treatments with currently used starting dosages per day compared with placebo (reference line through 0). ○ mean, 95% confidence interval, TDS transdermal system, VAS visual analogue scale.

Among adverse events, gastrointestinal side effects were most frequently reported. Only transdermal oxybutynin 3.9 mg/d showed similar gastrointestinal profiles to placebo (Figure 2). Ocular/visual adverse events were similar across the various antimuscarinics when starting dosages were used (Figure S3). This was also observed for urinary tract related (Figure S4), neurological (Figure S5), cardiac (Figure S6), and respiratory tract related (Figure S7) adverse event profiles. Dermatological adverse events were insignificant with oral drug administration but a worse profile was found with transdermal application (Figure S8).

Age, gender, and duration of treatment had no significant influence on adverse events.

## Discussion

### Main findings

Our network meta-analysis, which applies a transparent method, allows direct and indirect comparison of all currently used antimuscarinics, dosages, formulations and routes of administration. Moreover, our method provides an easy to grasp overview of side effects which is important since antimuscarinics for treating OAB are selected in clinical practice based on the trade off between drugs' efficacy and adverse events. The results of this analysis can be a valuable input for decision analytic models. Our approach also contributes to a recent call of the GRADE working group that the strength of evidence should be assessed considering trade-offs between effect and side effects. Overall adverse event profiles were similar for all antimuscarinics except for oxybutynin orally administered when comparing currently used starting dosages. In addition, except for trospium chloride, we found a positive and significant dose-adverse event relation for the antimuscarinics.

### Findings in the context of existing evidence

In respect to reported adverse events, our data confirm results of various previous systematic reviews [7]–[13]. Dry mouth was consistently the most common complaint [7], [8], [10], [12], [13], and this is in line with our findings that gastrointestinal adverse events were most frequently reported. In our study, most antimuscarinics had similar ocular/visual adverse events to placebo when their lower available dosage was used. This confirms the results of Chapple et al. [12] regarding blurred vision. Although the inhibitory effect of antimuscarinics on detrusor muscle contraction could theoretically cause or aggravate voiding difficulty and urinary retention, urinary tract related adverse events were not different between antimuscarinics and placebo. This is supported by a randomised, placebo-controlled trial in men with bladder outlet obstruction showing that antimuscarinic treatment did not adversely affect urinary function in this high risk group of patients [87]. Central nervous system (CNS) adverse events are of particular concern as muscarinic receptors are prominent in the CNS and play an important role in memory, vigilance, problem solving, stimulus and response processing [101]. Perry et al. [102] found an increased occurrence of Alzheimer's disease (AD) related to a prolonged antimuscarinic exposure in patients with Parkinson's disease raising the concern that chronic antimuscarinic treatment may increase the risk of AD or accelerate AD pathogenesis. Notwithstanding the fact that cognitive impairment traditionally has not been evaluated in antimuscarinics' trials, CNS adverse events were rare and we found an overall neurological adverse event profile for most antimuscarinics similar to placebo in our study. Cardiac adverse events, particularly the increase of heart rate and prolongation of the QT interval associated with polymorphic ventricular tachycardia (or torsade de pointes), can be serious, and the antimuscarinic agent terodiline was withdrawn from the market because of an association with QT prolongation [103]. However, there is no evidence from our data to suggest that the currently used antimuscarinics increase the risk of cardiac adverse events when administered at the recommended therapeutic dosages. Respiratory tract related adverse events were negligible. Dermatological adverse events were insignificant with oral drug administration but in accordance with previous meta-analyses [12], [13] a worse profile was found with transdermal application.

### Strength and weaknesses

Our network meta-analytic approach provides valuable additional information to conventional systematic reviews [7]–[13]. We performed a joint assessment of all reported adverse events of all currently prescribed antimuscarinics while by including a dummy variable, we maintained the comparison of two treatments for each of the studies. That the overall adverse event profiles of each antimuscarinic can be summarized in one simple graph (Figure 1) providing all relevant information at a glance is another strength of our approach. We believe that this information is useful for health authorities, policy makers, physicians, and patients alike. As estimates of the within study variance of the adverse event scores were not available, a customary network analysis investigating heterogeneity was not possible. In fact, we assumed heterogeneity by allowing the outcome parameter to have a variance comprising the between as well as the within study variance. Eleven studies used a crossover design. Dependent on the within-subject correlation, the variance of the within-subject comparison of the adverse event score will be lower than the between-subject comparison [104]. As the weights are inversely proportional with the variance, this would mean that the weights of the crossover studies should be increased. We performed a sensitivity analysis increasing the weights of studies with a crossover design with a factor two, showing only minimal differences with the presented results. Although our figures summarize the adverse event profiles of the antimuscarinics, it is unclear how predictive the mean values are for an individual patient.

We categorized the antimuscarinics based on the daily dosage used in clinical practice, but differences in medication frequency and formulations were not considered. Only for tolterodine a reasonable number of studies with both immediate and extended release formulations were available. However, using our meta-analytic approach, the influence of the release on the occurrence of adverse events could easily be investigated by entering an interaction term with treatment into the regression model. Almost all studies published are fixed dose trials and we excluded the few studies that did not clearly specify the dose regarding adverse events. This is an important limitation since in clinical practice the dose of a drug is often titrated and flexible dose studies tend to report fewer adverse events. Duration of treatment had no significant influence on the adverse event profiles but absence of evidence is not evidence of absence. None of the trials reported whether and how many patients had two or more adverse events. Therefore, we had to assume that the occurrence of an adverse event was independent of the presence of another, although this might be more complex in clinical reality. Moreover, policy and completeness of adverse event reporting changed during the last 20 years and differ between the trials. The impact of this variability on the results is difficult to determine, but under-reporting of adverse events, particularly in earlier trials, is likely. It might be argued, that the grading of adverse events should be based on patients' opinion, taking into account that patients' and physicians' values and preferences can differ widely [105]. Basing the grading system on patients' preferences would imply an interview in a representative and extensive sample. However, this would require an additional study that certainly should be performed to include patients' grading in this analysis. As the decision for a specific antimuscarinic treatment is usually taken by the physician who is supposed to have a deliberate overview of the manifestations of a specific adverse event, we decided to base our grading of adverse events on the consensus of 10 independent experts.

To grade the strength of recommendation, the quality of the evidence should be assessed. Although only randomized trials are included (i.e. the highest grade) the quality of evidence should be downgraded because in most studies the reporting of adverse events will be imprecise and the probability of reporting bias is presumable.

### Implications for research

We believe that our method provides a useful overview of benefits and downsides of various medications prescribed in a particular clinical area. To us, the method is of most value for researchers commissioned to provide comprehensive summaries and decision-analytic models to guide decisions of health authorities such as the Centres of Reviews and Disseminations (CRDs) in the UK or similar institutions in Germany and the United States of America. We would like to point out that this approach is equally applicable to the assessment of treatment effects. From a decision-making point of view there are new opportunities that could be explored. For example, adding the patients' perspective regarding the burden of adverse events and the minimum expected beneficial effect could be used as benchmark criteria against which the optimal regimen could be selected. Finally, data of the form provided in this article are an ideal starting point for various cost-effectiveness analyses.

### Implications for practice

Although antimuscarinics are generally regarded to be well tolerated, non-adherence to medication represents a major challenge. In a community setting, up to 40% of patients may discontinue antimuscarinic treatment due to adverse events [106]. As shown in previous systematic reviews [7]–[13], all currently used antimuscarinics have similar efficacy for the usually prescribed starting dosages (Figure S1) and this view is also supported by the NICE guideline [107]. Thus, differences in adverse event profiles should guide the choice for treatment. We showed that darifenacin 7.5 mg/d, fesoterodine 4 mg/d, transdermal oxybutynin 3.9 mg/d, propiverine 30 mg/d, solifenacin 5 mg/d, tolterodine 4 mg/d, and trospium chloride 40 mg/d (or 60 mg/d) had similar overall adverse event profiles and seem to be equivalent first choice drugs to start the treatment of OAB. In case these first choices are ineffective, dose escalation or changing to another antimuscarinic appears to be reasonable. Oxybutynin dosages of ≥10 mg/d are of questionable value in the current treatment of OAB because of unfavorable adverse event profiles.

### Conclusions

We propose a generally applicable method that summarizes adverse events in an easy to grasp way using a transparent network meta-analysis which incorporates all available information from clinical trials while fully maintaining randomization. Darifenacin 7.5 mg/d, fesoterodine 4 mg/d, transdermal oxybutynin 3.9 mg/d, propiverine 30 mg/d, solifenacin 5 mg/d, tolterodine 4 mg/d, and trospium chloride 40 mg/d (or 60 mg/d) seem to be equivalent first choice drugs to start the treatment of OAB, whereas oral oxybutynin dosages of ≥10 mg/d have less favorable adverse event profiles.

## Supporting Information

Figure S1 Efficacy of different antimuscarinics (starting dosages) compared to placebo (reference line through 0) according to Chapple et al, Eur Urol 2008;54:543‐62 and Staskin et al, Eur Urol 2009;55:e49‐50.
(TIF)

Figure S2 Flow diagram of literature searches and results.
(TIF)

Figure S3 Ocular/visual adverse event profiles (from 45 trials) of different antimuscarinic treatments with currently used starting dosages per day compared with placebo (reference line through 0). **○** mean, 95% confidence interval, TDS transdermal system, VAS visual analogue scale.
(TIF)

Figure S4 Urinary tract related adverse event profiles (from 37 trials) of different antimuscarinic treatments with currently used starting dosages per day compared with placebo (reference line through 0). **○** mean, 95% confidence interval, TDS transdermal system, VAS visual analogue scale.
(TIF)

Figure S5 Neurological adverse event profiles (from 52 trials) of different antimuscarinic treatments with currently used starting dosages per day compared with placebo (reference line through 0). **○** mean, 95% confidence interval, TDS transdermal system, VAS visual analogue scale.
(TIF)

Figure S6 Cardiac adverse event profiles (from 22 trials) of different antimuscarinic treatments with currently used starting dosages per day compared with placebo (reference line through 0). **○** mean, 95% confidence interval, TDS transdermal system, VAS visual analogue scale.
(TIF)

Figure S7 Respiratory tract related adverse event profiles (from 20 trials) of different antimuscarinic treatments with currently used starting dosages per day compared with placebo (reference line through 0). **○** mean, 95% confidence interval, TDS transdermal system, VAS visual analogue scale.
(TIF)

Figure S8 Dermatological adverse event profiles (from 21 trials) of different antimuscarinic treatments with currently used starting dosages per day compared with placebo (reference line through 0). **○** mean, 95% confidence interval, TDS transdermal system, VAS visual analogue scale.
(TIF)

Data S1 Additional information kindly provided by authors of eligible trials that reported insufficient data.
(DOC)

Data S2 Database of included trials.
(XLS)
