# Supplementary figures and images for: Adverse Event Assessment of Antimuscarinics for Treating Overactive Bladder: A Network Meta-Analytic Approach
Source: PLoS One. 2011 Feb 23;6(2):e16718. doi: 10.1371/journal.pone.0016718 (PMC3044140; doi:10.1371/journal.pone.0016718)

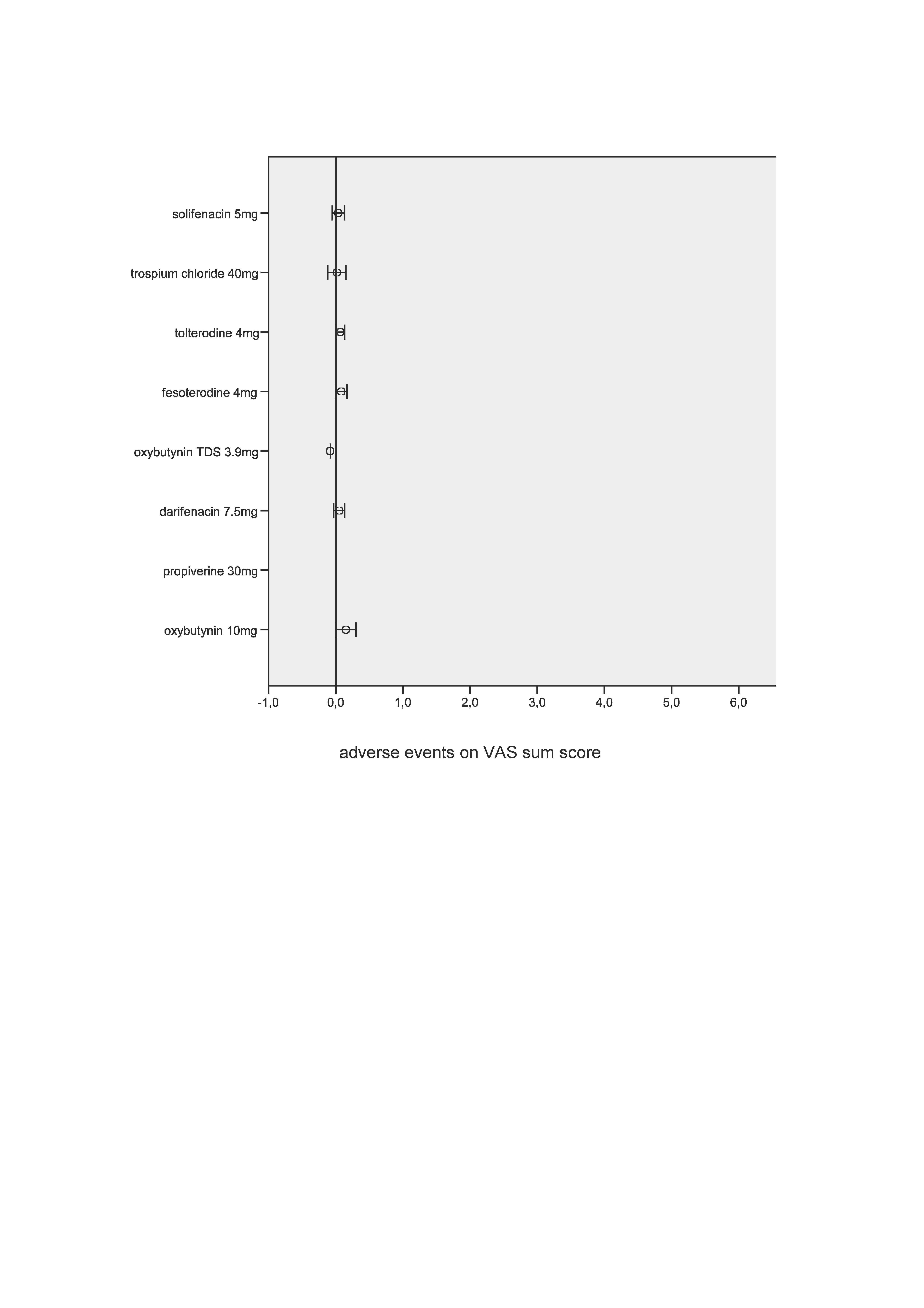

Supplement: Figure S3 — Ocular/visual adverse event profiles (from 45 trials) of different antimuscarinic treatments with currently used starting dosages per day compared with placebo (reference line through 0). ○ mean, 95% confidence interval, TDS transdermal system, VAS visual analogue scale. (TIF) [file pone.0016718.s003.tif]

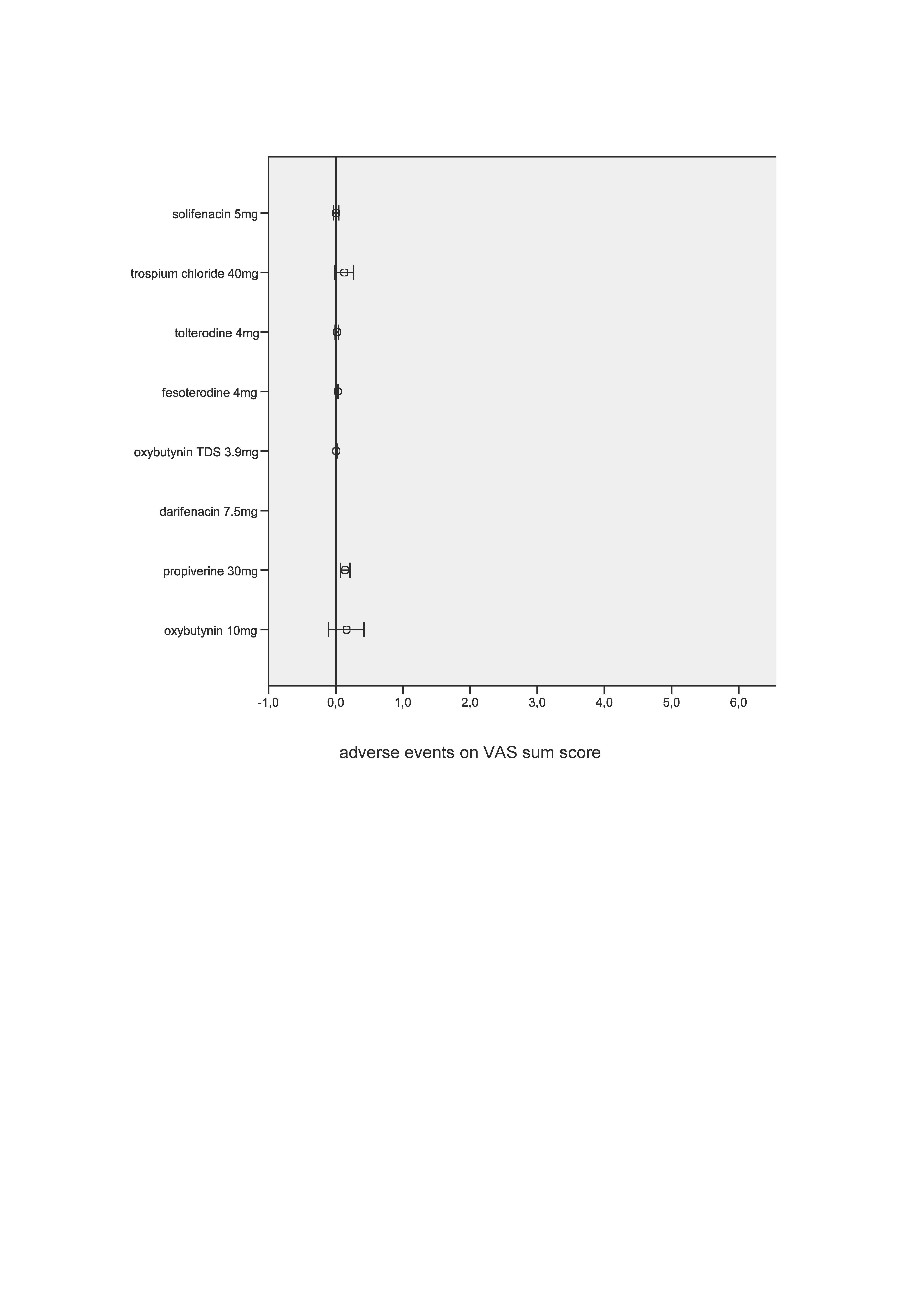

Supplement: Figure S4 — Urinary tract related adverse event profiles (from 37 trials) of different antimuscarinic treatments with currently used starting dosages per day compared with placebo (reference line through 0). ○ mean, 95% confidence interval, TDS transdermal system, VAS visual analogue scale. (TIF) [file pone.0016718.s004.tif]

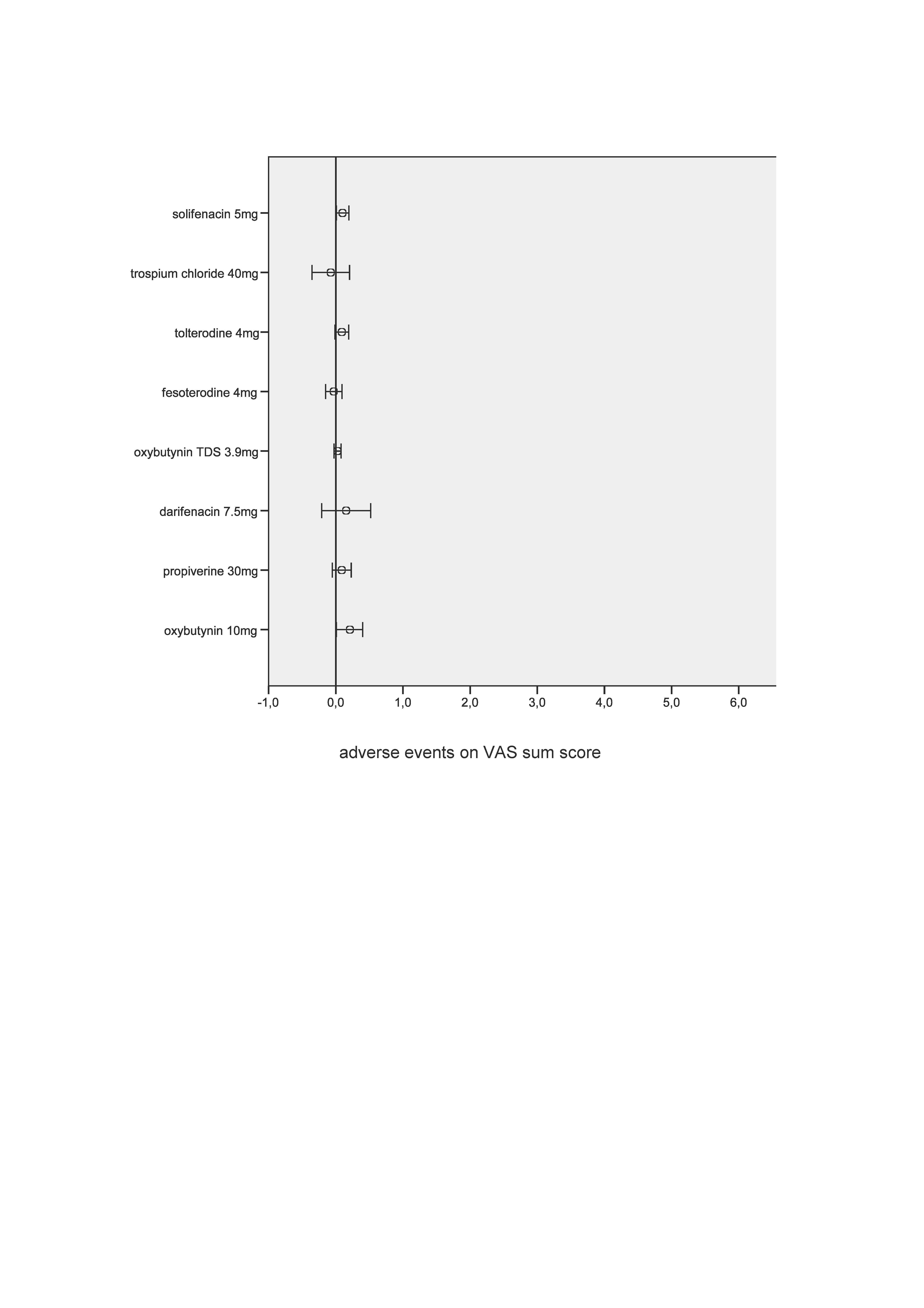

Supplement: Figure S5 — Neurological adverse event profiles (from 52 trials) of different antimuscarinic treatments with currently used starting dosages per day compared with placebo (reference line through 0). ○ mean, 95% confidence interval, TDS transdermal system, VAS visual analogue scale. (TIF) [file pone.0016718.s005.tif]

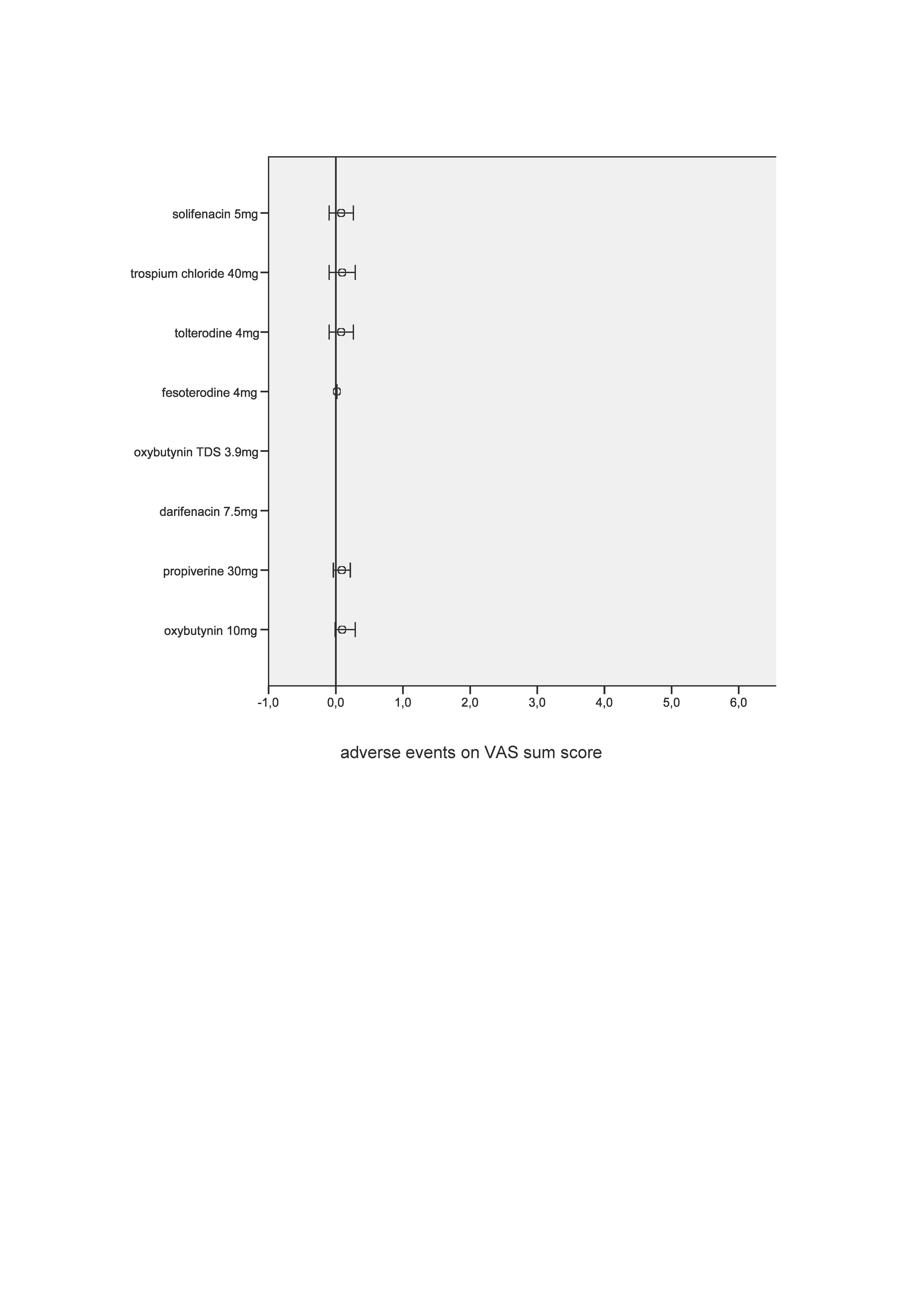

Supplement: Figure S6 — Cardiac adverse event profiles (from 22 trials) of different antimuscarinic treatments with currently used starting dosages per day compared with placebo (reference line through 0). ○ mean, 95% confidence interval, TDS transdermal system, VAS visual analogue scale. (TIF) [file pone.0016718.s006.tif]

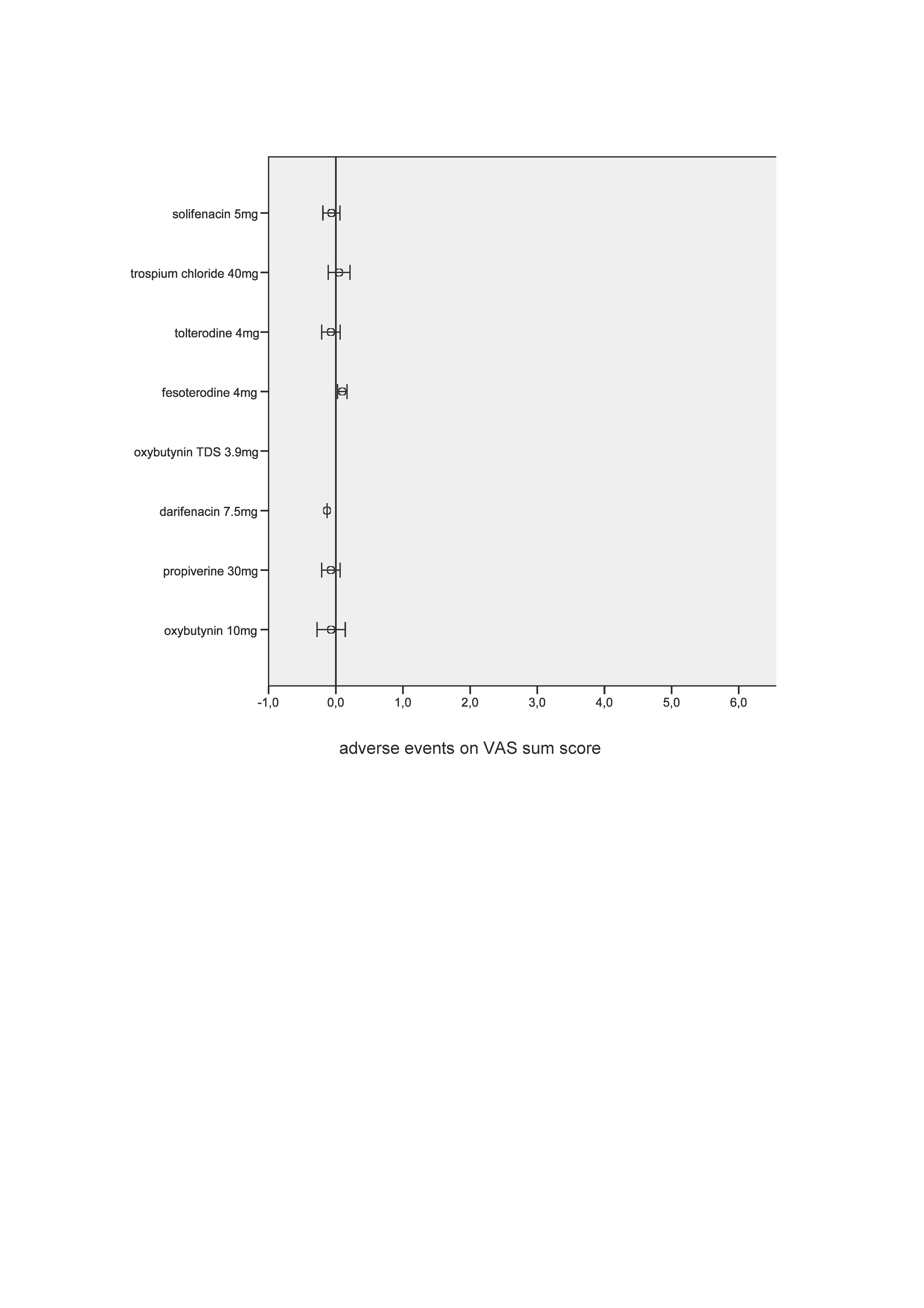

Supplement: Figure S7 — Respiratory tract related adverse event profiles (from 20 trials) of different antimuscarinic treatments with currently used starting dosages per day compared with placebo (reference line through 0). ○ mean, 95% confidence interval, TDS transdermal system, VAS visual analogue scale. (TIF) [file pone.0016718.s007.tif]

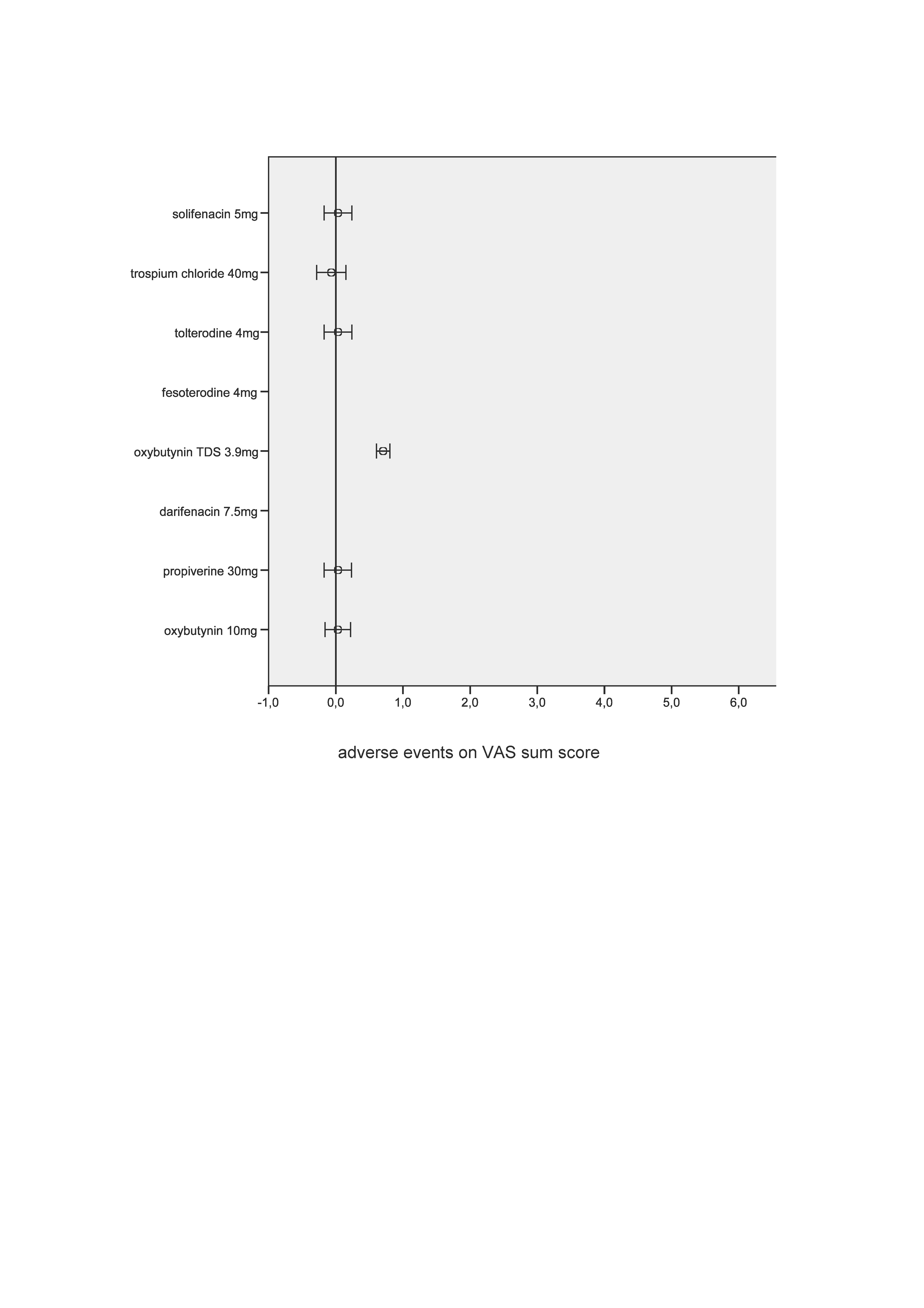

Supplement: Figure S8 — Dermatological adverse event profiles (from 21 trials) of different antimuscarinic treatments with currently used starting dosages per day compared with placebo (reference line through 0). ○ mean, 95% confidence interval, TDS transdermal system, VAS visual analogue scale. (TIF) [file pone.0016718.s008.tif]
