# Supplementary material for: Adverse Event Assessment of Antimuscarinics for Treating Overactive Bladder: A Network Meta-Analytic Approach
Source: PLoS One. 2011 Feb 23;6(2):e16718. doi: 10.1371/journal.pone.0016718 (PMC3044140; doi:10.1371/journal.pone.0016718)
Supplement: Data S1 — Additional information kindly provided by authors of eligible trials that reported insufficient data. (DOC) [file pone.0016718.s009.doc]

Madersbacher H, Halaska M, Voigt R, Alloussi S, Hofner K (1999) A placebo-controlled, multicentre study comparing the tolerability and efficacy of propiverine and oxybutynin in patients with urgency and urge incontinence. BJU Int 84: 646-651

| Adverse event | Propiverine IR 15mg tid (n=149)  n | Oxybutynin IR 5mg bid (n=145)  n | Placebo (n=72)  n |
| --- | --- | --- | --- |
| fatigue | 27 | 34 | 9 |
| somnolence | - | - | - |
| sedation | - | - | - |
| insomnia | - | - | - |
| confusion | 4 | 6 | 0 |
| cognitive impairment | - | - | - |
| depression / lethargy | 2 | 0 | 0 |
| dizziness / vertigo | 17 | 14 | 9 |
| headache | 2 | 4 | 2 |

Homma Y, Paick JS, Lee JG, Kawabe K (2003) Clinical efficacy and tolerability of extended-release tolterodine and immediate-release oxybutynin in Japanese and Korean patients with an overactive bladder: a randomized, placebo-controlled trial. BJU Int 92: 741-747

| Adverse event | Tolterodine ER 4mg qd (n=239)  n | Oxybutynin IR 3mg tid (n=244)  n | Placebo (n=122)  n |
| --- | --- | --- | --- |
| fatigue | 3 | 1 | 0 |
| sedation | - | - | - |
| insomnia | 2 | 1 | 0 |
| confusion | - | - | - |
| cognitive impairment | - | - | - |
| depression / lethargy | - | - | - |

Hill S, Khullar V, Wyndaele JJ, Lheritier K (2006) Dose response with darifenacin, a novel once-daily M3 selective receptor antagonist for the treatment of overactive bladder: results of a fixed dose study. Int Urogynecol J Pelvic Floor Dysfunct 17: 239-247

| Adverse event | Darifenacin 7.5mg qd (n=108)  n | Darifenacin 15mg qd (n=107)  n | Darifenacin 30mg qd (n=115)  n | Placebo  (n=109)  n |
| --- | --- | --- | --- | --- |
| fatigue | 3 | 3 | 3 | 2 |
| somnolence | 1 | 1 | 1 | 0 |
| sedation | - | - | - | - |
| insomnia | 0 | 0 | 1 | 0 |
| confusion | - | - | - | - |
| cognitive impairment | - | - | - | - |
| depression / lethargy | 1 | 1 | 0 | 0 |
| dizziness / vertigo | 1 | 2 | 0 | 0 |

Jünemann KP, Hessdorfer E, Unamba-Oparah I, Berse M, Brunjes R, et al. (2006) Propiverine hydrochloride immediate and extended release: comparison of efficacy and tolerability in patients with overactive bladder. Urol Int 77: 334-339

| Adverse event | Propiverine IR 15mg bis (n=395)  n | Propiverine ER 30mg qd (n=391)  n | Placebo (n=202)  n |
| --- | --- | --- | --- |
| fatigue | 4 | 1 | 0 |
| somnolence | 1 | 0 | 1 |
| sedation | - | - | - |
| insomnia | 1 | 1 | 0 |
| confusion | 1 | 0 | 0 |
| cognitive impairment | - | - | - |
| depression / lethargy | - | - | - |
| dizziness / vertigo | 7 | 8 | 3 |
| headache | 8 | 6 | 1 |
